# Establishing event-based surveillance system in Nigeria: a complementary information generating platform for improved public health performance, 2016

**DOI:** 10.11604/pamj.2022.42.63.29621

**Published:** 2022-05-24

**Authors:** Rabi Usman, Lawal Bakare, Ubong Okon Akpan, Mahmood Dalhat, Augustine Olajide Dada, Ifeanyi Okudo, Robinson Nnaemeka Nnaji, Muhammad Shakir Balogun, Saheed Gidado, Patrick Nguku

**Affiliations:** 1 Nigeria Field Epidemiology and Laboratory Training Program, Abuja, Nigeria,; 2 Epid Alert, Lagos, Nigeria,; 3 Department of Public Health Nigeria, Police Medical Service, Benue State, Nigeria,; 4 African Field Epidemiology Network (AFENET), Abuja, Nigeria,; 5 World Health Organization, Abuja, Nigeria,; 6 Nigeria Centre for Disease Control, Abuja, Nigeria,

**Keywords:** Event-based surveillance system, integrated disease surveillance and response system (IDSR), awareness, Nigeria

## Abstract

**Introduction:**

event-based surveillance (EBS) is a surveillance method involving systematic and prompt data collection on incidents of public health importance, and complements the current indicator-based surveillance system and the Integrated Disease Surveillance and Response System (IDSR). It also promotes a rapid assessment and response to public health emergencies in Nigeria, although there is a lack of information regarding the status of EBS among Public Health Stakeholders in Nigeria; hence our study aimed to assess the awareness, availability, and utility of EBS among Nigerian public health stakeholders.

**Methods:**

we conducted a cross-sectional study to assess the awareness, availability, functionality, and utilization of EBS in the 36 States in Nigeria, plus the Federal Capital Territory (FCT). We interviewed 53 stakeholders in disease surveillance and response using a self-administered, semi-structured questionnaire to obtain responses on the awareness of the event-based surveillance system, availability, and functionality. We also assessed the common structures used to report health-related events and the availability of minimum requirements for an event-based surveillance system. We performed descriptive statistics for the data obtained.

**Results:**

the majority of respondents were males and 37.7% were disease surveillance and notification officers (DSNOs). Awareness of EBS was poor with about half, 49% of the respondents reported hearing of EBS, but only 17% described it correctly. The overall level of availability of the EBS reporting structure was inadequate, 28.2% and poorly utilised in the States.

**Conclusion:**

the awareness, availability, and utilization of event-based surveillance systems are low in Nigeria. The government should improve the feasibility and utility of EBS in the States to enhance early disease detection and response.

## Introduction

Event-based surveillance is the systematic and rapid collection of data regarding incidents that could pose a public health danger [1]. Rumors and other ad-hoc reports can be distributed via structured monitoring structures and informal networks such as the media, health workers, and community reports. It also contains records of incidents relating to the prevalence of disease in the community and the occurrence of disease in humans, such as clusters of cases of a disease, abnormal disease patterns, or sudden unexplained deaths in humans as reported by health workers and other community key informants, including diseases and deaths in animals, contaminated food or water, and environmental hazards [1,2]. According to the World Health Organization, event-based surveillance complements the IDSR [1,3] and will significantly improve the sensitivity of the surveillance system [2]. Both systems should be considered integral parts of a unified national surveillance scheme [1]. When it comes to detecting outbreaks and major public health events promptly, IDSR can fall short [1]. Furthermore, the systems are unsuitable for detecting uncommon but high-impact outbreaks (Ebola, Avian Influenza), as well as emerging and unknown diseases [4].

However, an event-based surveillance system would play a key role in ensuring early detection and prompt response. The IDSR acknowledges and charges state epidemiologists and disease surveillance and notification officers (DSNOs) with the responsibility of ensuring early disease detection and response [5]. Still, an event-based monitoring system requires an event assessment team to assist with preliminary event confirmation and, where skills and resources exist, initial event assessment and a rapid response capacity for preliminary investigation [1]. Following the recommendation by global platforms such as the World Health Organization (WHO), the European Centers for Disease Control (ECDC), and the United States Centers for Disease Control (US-CDC) on the adoption of event-based surveillance at the country level, especially for member states with poor health surveillance systems, which would reinforce the framework for an effective and efficient response to emerging infectious diseases, developing countries like Nigeria are yet to optimize the usefulness of EBS in response to emerging infectious diseases. This may be attributed to the poorly known benefit of early warning systems, poor understanding of EBS, incompletely organized, and poorly developed standard operating procedures [6]. Considering that diagnosis, monitoring, active reporting, timely healthcare delivery, safe and effective treatment to fight infectious diseases are inadequate and poorly implemented, the states need to develop and implement a robust event-based surveillance system to strengthen active surveillance of emerging infectious diseases. To this end, we carried out research to assess the awareness, availability, and utility of EBS among Nigerian public health stakeholders.

## Methods

**Study design:** the study design was a descriptive cross-sectional study with a quantitative method approach.

**Setting:** the study was conducted during the annual surveillance review meeting organised by the Nigeria Centre for Disease Control (NCDC) in Ibadan, Oyo State, between 20^th^ and 24^th^ March 2016. This meeting was attended by participants across the 36 states plus the Federal Capital Territory (FCT).

**Sampling technique:** consecutive sampling was used to enroll participants in the study.

**Participants:** this study was conducted among disease surveillance and notification officers (DSNOs), state epidemiologists, laboratory specialists, and public health experts who attended the annual surveillance review meeting organised by Nigeria Center for Disease Control. The eligibility criteria are any surveillance officer attending the annual disease surveillance review meeting in Ibadan, Nigeria.

**Study size:** all the participants who attended the meeting and provided written consent were enrolled in the study; hence sample estimation was not done. Fifty-three participants were enrolled in the study.

**Data collection:** a semi-structured, self-administered questionnaire was used to obtain information on sociodemographic characteristics of respondents, awareness of EBS, the meaning of EBS, reporting of health and health-related events using the EBS structure, availability of channels used to report EBS, and its functionality (Annex 1). The questionnaire used for the study was adapted from the WHO event-based surveillance guideline [7]. It was piloted among Nigeria field epidemiology and laboratory training programme (NFELTP) residents working in the surveillance unit of the Nigeria Centre for Disease Control. A final review was done following the outcome of the pilot study by three epidemiologists.

**Data entry and analysis:** data were checked for completeness before entry, cleaned, and analysed using Epi-Info 7.1. Descriptive statistics were computed for quantitative variables to calculate frequencies and proportions. We determined the level of awareness among the study participants using having heard about EBS and the ability to describe EBS correctly. The total score was calculated, and the average percentage value was derived and categorised as less than 50% to be poor awareness; 50%-80% were considered good awareness, while above 80% was regarded as excellent awareness. The availability of EBS was assessed across the thirty-six states plus the FCT of Nigeria using the presence of an EBS reporting structure and the use of email as a method of event reports. The average scores were calculated with values converted into percentages and categorised as less than 50% as grossly inadequate, 50%-80% were considered as slightly adequate while above 80% was considered an adequate EBS system present in the states. Utility of EBS in Nigeria was assessed using the number of states with available and functional emergency preparedness response (EPR) team, drugs, personal protective equipment (PPEs), information, education and communication (IEC) materials prepositioned, and the availability of emergency funds for outbreaks response that can be accessed within 24hours of the start of an outbreak. The cumulative percentage was calculated and categorized as less than 50% considered a poor utility, 50-80% regarded as a moderately good utility, while above 80% considered a good utility of the EBS system. We presented our results using tables, maps and structured the report following the Strobe statement [8].

### Ethical consideration

**Research assistants**: research assistants were four epidemiologists trained on the survey tools, administering the consent form, and supporting the participants to complete the survey.

**Ethical approval:** the Ministry of Health Research Ethics Committee approved the research with approval number ZSHREC02032016. Permission was sought from the Nigeria Centre for Disease Control (NCDC) as the convener of the annual review meeting. Participants were aware of the study, and participation was voluntary.

**Informed consent:** before receiving the survey questionnaire, written informed consent was administered to all participants in English. The participants were not exposed to harm due to their participation at any point in time. They were informed of their ability to withdraw participation at any time during the completion of the questionnaire. The consent paper given to participants contained all the information required to make an informed decision, including all elements of informed consent as required by the National Code of Health Research Ethics (Code 35). This guideline was strictly followed during the study.

**Confidentiality:** confidentiality of collected information was maintained by using unique non-personal identifier codes for the participants. The completed questionnaire was kept under lock and key. Data stored in the computer were passworded and only assessed by the principal investigator and the authors directly involved in the data management and analysis.

## Results

The sociodemographic characteristics of respondents are summarized in Table 1. Fifty-three (53) respondents from the 36 states plus the FCT participated in the study. Most of the respondents were males, 39 (73.6%). More than one-third, 28 (37.7%), were state disease surveillance and notification officers (state DSNOs), and the least were assistant state DSNOs, 2 (3.8%).

**Table 1:** distribution of EBS respondents by sociodemographic characteristics, Nigeria 2016

Characteristic N=53	Frequency	Percent
**Gender**		
Male	39	73.6
Female	14	26.4
**Designation**		
State epidemiologist	20	37.7
Deputy state epidemiologist	3	5.7
State DSNO*	28	52.8
Assistant state DSNO	2	3.8

* DSNO: disease surveillance and notification officer; EBS: event-based surveillance

**Awareness of event-based surveillance:** forty-nine percent (49%) of respondents have heard of the event-based surveillance system, but only 9 (17%) described event-based surveillance correctly, as summarized in Table 2. The level of awareness among the study participants was poor (33%).

**Table 2:** respondents' awareness and correct description of EBS in Nigeria, 2016

Characteristic N=53	Frequency	Percent
**Ever heard of EBS**		
Yes	26	49.0
No	27	51.0
**Correctly described EBS**		
Yes	9	17.0
No	44	83.0
Average (yes)	35	33.0

EBS: event-based surveillance

**Figure 1:**
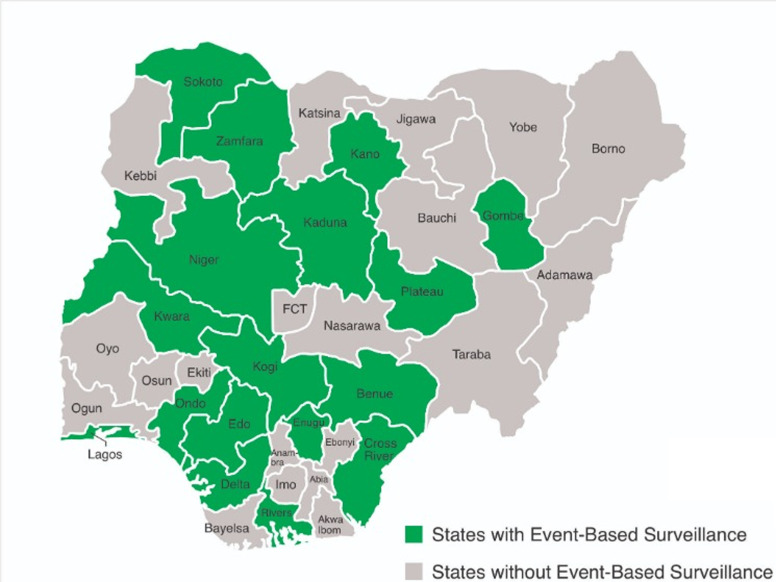
map of Nigeria showing States with an event-based surveillance system in green

**Availability of event-based surveillance and EBS reporting structures:** seventeen (46%) out of the 36 states (plus FCT) have an event-based reporting structure. This is through phone calls, SMS, DSNOs, and the use of community informants. Nine states (24%) reported getting information through emails. WhatsApp as a reporting channel was found in 3 states (8.1%); such reports were random and did not occur routinely. None of the states in Nigeria use Twitter, Facebook, or other social media platforms to report health-related events to the authorities concerned. The overall level of availability of the EBS system reporting structure in Nigeria is 28.2% which is inadequate (Figure 1).

**Utility of event-based surveillance in Nigeria:** twelve (32.4%) states have an emergency preparedness and response (EPR) team. Emergency preparedness and response team was found to be functional in 10 (27.0%) states and have medications, personal protective equipment (PPEs), and information, education, and communication (IEC) materials repositioned for deployment in the event of a disease outbreak. None of the states has emergency funds that can be mobilized within 24 hours for managing disease outbreaks. Resources are usually mobilized while the outbreak is ongoing, and these usually come late. Findings showed that the utility of EBS is poorly utilized in Nigeria, 24.9% (Table 3).

**Table 3:** availability of minimum requirements for EBS in Nigeria, 2016

Characteristic N=37	Frequency	Percent
**EPR team available**		
Yes	17	45.9
No	20	54.1
**EPR team functional**		
Yes	10	27.0
No	27	73.0
**Drugs, PPEs, IEC materials prepositioned**		
Yes	10	27.0
No	27	73.0
**Emergency funds for outbreaks are available**		
Yes	0	0.0
No	37	100.0
Average (Yes)	37	24.9

EPR: emergency preparedness and response; PPE: personal protective equipment; IEC: information, education and communication

## Discussion

This study shows poor awareness of the EBS system in Nigeria. This is because the EBS system of reporting has not been fully established and standardized in the country. Another reason for this may be that emphasis has been on reporting diseases and other health events using the IDSR platform, with little awareness among disease surveillance officers and the general population on using EBS to complement IDSR. Disease outbreaks and health events reported using phone calls and SMS to the DSNOs and community informants were available in some states in Nigeria. States like Lagos and Kano have dedicated call centers for reporting unusual events to health authorities [9]. For other states, the major challenge is the lack of dedicated personnel and funds to monitor the call centers and other EBS platforms. Social media channels such as WhatsApp, Twitter, Facebook, and Instagram are not regular channels used for reporting diseases in Nigeria as at the time this study was conducted.

An EBS system has some minimum requirements for it to function effectively [1]. Only one-third of the states in the country have an emergency preparedness and response (EPR) committee responsible for coordination and mobilization of resources during outbreaks. It is important to note that outbreak response cannot go well if there are no adequate funds. No state in Nigeria reported having emergency funds dedicated to outbreak response that can be accessed within 24 hours as at the time of this study. Lack of funds for outbreak response will lead to a delayed response, with the resultant increase in disease morbidity and mortality. EBS reporting was not common in Nigeria until the Ebola outbreak in 2014 when communication centers were launched, and 24-hour toll-free lines were rolled out in Lagos State to report suspected Ebola cases. Presently, a publicly available toll-free number is dedicated to receiving and making calls and receiving messages at the connect center at the Nigeria Center for Disease Control, where a communications officer and call center personnel are responsible for monitoring phone calls, text, and WhatsApp messaging. All channels of communication in the connect center are open to the public and monitored 24/7. Also, there is Tatafo, a data mining and analytical tool used to retrieve hits from over a thousand media sources. It was launched by the University of Maryland Baltimore (UMB) in partnership with Nigeria Center for Disease Control (NCDC). Tatafo retrieves trending information through configured keywords coined from forty-one (41) notifiable diseases, deaths, and health events [10].

**Limitation:** the participants for this study were selected using convenient sampling; hence our findings are not generalisable to the population.

## Conclusion

There is low awareness of the event-based surveillance system in Nigeria. The availability of EBS across States in Nigeria is inadequate, while the utility of EBS in Nigeria is poor. For a fully functional event-based surveillance system that can detect health and health-related events, the government will need to ensure the following: increase awareness and strengthen the sensitivity of EBS through training of people in and outside the health system (e.g health workers, media, village leaders, community members etc.); provide a 24-hour telephone hotline, fax or email to receive reports at local government areas (LGA) and State levels for reporting health events, provide financial support and human resource capacity to ensure the sustainability of EBS system.

### What is known about this topic

- Event-based surveillance complements integrated disease surveillance and response;
- Event-based surveillance is an emerging area in the surveillance of infectious diseases.

### What this study adds

- There is low awareness of event based surveillance in Nigeria;
- Only one-third of States in the country have an EPR committee responsible for coordinating and mobilising resources during outbreaks;
- The basic infrastructure for EBS was found to be available but inadequate across the States in Nigeria

## Annex

**Annex 1**: questionnaire on the availability and funtionality of event-based surveillance in Nigeria (PDF-357KB)
